# Simultaneous early ovarian and endometrial cancer treated conservatively with spontaneous pregnancy

**DOI:** 10.1186/1757-2215-6-59

**Published:** 2013-08-21

**Authors:** David Atallah, Joelle Safi, Nadine el Kassis, Roman Rouzier, Georges Chahine

**Affiliations:** 1 Obstetrics and Gynecology, Hôtel Dieu de France University Hospital, Saint Joseph University, Beirut, Lebanon; 2 Institut Curie comprehensive cancer center, Paris, France; 3 Medical Oncology, Hôtel Dieu de France University Hospital, Saint Joseph University, Beirut, Lebanon

**Keywords:** Conservative treatment, Endometrial, Ovarian cancer, Pregnancy, Synchronous

## Abstract

**Introduction:**

Young cancer patients increasingly request fertility sparing alternatives to their cancer treatments, which they should be offered when allowed so by the risk-benefit balance and after obtaining informed consent.

**Case presentation:**

Here, we report the case of a 25 year-old nulliparous patient who presented with a synchronous endometrioid ovarian and endometrial carcinoma. She was able to conduct a full-term spontaneous pregnancy after conservative surgical treatment followed by adjuvant chemotherapy and hormonal treatment. Fertility sparing treatment is feasible in selected cases of synchronous ovarian and endometrial cancers. Thorough follow-up remains mandatory.

**Conclusion:**

This case demonstrates some interesting and unique features of synchronous ovarian and endometrial cancers since it resulted in a spontaneous pregnancy and normal delivery.

## Background

The gynecologic oncologist is in a unique position to provide young gynecologic cancer patients with fertility-sparing surgical alternatives when allowed so by tumor stage and histologic differentiation, while still offering the best curative treatment. Fertility sparing surgery for epithelial ovarian cancer as well as endometrial carcinoma has been abundantly described in the literature, in small series and retrospective cohorts [1-4]. The synchronous occurrence of both tumors, especially in young patients, is equally well-reported [5,6]. The conservative management of early ovarian and endometrial cancer is possible in certain histologic profiles. This is followed by a spontaneous pregnancy and normal delivery. To date, our own search in the English literature revealed that our case is the first reported one of conservatively treated synchronous endometrial and ovarian cancer followed by a successful pregnancy.

## Case presentation

A 25 year-old nulliparous woman was addressed to our center, Hôtel Dieu de France University Hospital, few weeks after being operated by laparoscopy of an 11 cm ruptured left adnexal cystic mass on May 2010. Pathology results showed an endometrioid grade I ovarian adenocarcinoma.

She was subsequently scheduled for a conservative surgery that included a total abdominopelvic exploration, left adnexectomy, peritoneal washings, random biopsies, infra-gastric omentectomy, resection of trocars insertion sites and bilateral ilio-obturator as well as para-aortic lymph node dissection reaching the left renal vein. An endometrial curettage was also carried out and later showed a well-differenciated endometrioid adenocarcinoma with complex hyperplasia. A frozen section at the time of surgery confirmed the absence of residual proliferating or viable tumoral cells in the left ovary with only necrotic granulomatous material left. Peritoneal cytology, omentectomy, lymph nodes (26 pelvic and 17 para-aortic) and trocar insertion sites were all negative for malignancy.

A month later, hysteroscopy with total surgical endometrectomy confirmed the total absence of myometrial involvement as the results came back with only complex hyperplasia with atypia. The patient went on and received six courses of carboplatin plus cyclophosphamide combined with six months of megestrol acetate and leuprolide acetate.

Histological controls, six months then a year later, obtained by hysteroscopy demonstrated the absence of residual hyperplasia, atypia or malignancy. She resumed menses after stopping megestrol and leuprolide.

She was scheduled for a control in May 2012 for a hysteroscopic control, but she turned out to be pregnant. She was followed up by sonograms during her pregnancy in order to rule out any recurrence. She had a normal delivery in Paris, on February 2013. Actually the patient is doing well with no evidence of disease.

## Conclusions

This case exposes both a synchronous occurrence of early-stage ovarian and endometrial carcinoma and the conservative approach undertaken.

Coexistence of carcinoma of the ovary and endometrium is not a rare event and has been reported in 5 to 10% of the cases from both origins [7], and as high as 25% in Walsh *et al*. [8], with no surgical or histological proof of whether it is a synchronous occurrence or a metastasis from one location to another. Synchronicity has generally been contemplated more often based on the demonstrated more favorable prognosis as well as the similar pathologic appearance, while molecular studies have been discordant. It has been suggested that embryologically similar tissues, such as those of the female genital tract, may be subject to a continuous or persistent carcinogenic effect with an increased susceptibility [2], possibly explaining synchronous or future development of carcinoma in preserved organs, i.e. in conservative treatment.

As a consequence, there is no established standard treatment, as it varies between the institutions. Additionally, synchronous tumors are frequently undiagnosed or understaged [9]. This is mainly due to the limited value of preoperative exams, but also to the fact that surgical exploration by laparoscopy is not systematic in endometrial cancer patients in order to verify the absence of ovarian involvement. Nevertheless, intra-operative assessment of ovarian pathology is fallible. Besides, the presence of occult malignancy found in grossly normal-appearing ovaries in the population of young women has already been reported by various authors [10].

Our patient was different in that she was first diagnosed with her ovarian tumor, her endometrial tumor being asymptomatic, and due to her age and endometrioid histology, an endometrial curettage was advocated and carried out to rule out endometrial malignancy.

Histologic features of these synchronous tumors are generally uncommon and particular to this population. These should elicit the suspicion of synchronous tumors with a very low incidence of poorly differentiated or grade 3 cancers, and a very low incidence of serous, mucinous, or clear-cell histotype of ovarian cancer, which otherwise would typically account for 70 to 80% of tumors [9]. Synchronous tumors are generally much more common in young patients less than 40 years old. Survival rates are generally very high, with an estimated recurrence rate at 5 years as low as 10% in women with cancers limited to the uterus and ovaries according to Zaino *et al.*[7]. The reason for the better than expected survival for these patients is not intuitively obvious. Hence it remains to date an observational pattern.

There are a number of studies reporting conservative treatment for ovarian as well as endometrial cancer.

Firstly, the actual gold standard for treating epithelial ovarian cancer (EOC) remains an adequate and complete operative staging with the double objective of adapting the therapeutic plan to the potential occult advanced disease and of estimating the impact on overall prognosis. Nonetheless, according to the 2007 guidelines of the American College of Obstetrics and Gynecology (ACOG), fertility-sparing surgery (FSS) for reproductive-age patients with invasive EOC is recommended for highly or moderately differentiated stage IA disease with favorable (non-clear-cell) histology [11]. The European Society for Medical Oncology (ESMO) also had a very similar statement in 2008 [12]. However, the level of evidence remains low, due to the limited number of studies and the small patient samples [1]. In a recent study by Kashima *et al*. [13], reproductive outcomes after FSS were found to be favorable in a study including 18 patients with FIGO stage IC epithelial ovarian cancer. Out of 10 patients who attempted to conceive, 7 singleton pregnancies were recorded for 5 women (50%) and all neonates were delivered at term. Overall, when combining their results with 7 other similar studies, namely Zanetta *et al.*[14], Schilder *et al.*[15], Morice *et al.*[16], Borgfeldt *et al.*[17], Park *et al.*[18], Kajiyama *et al.*[19], and Satoh *et al.*[20], 125 of 502 women in the combined cohort became pregnant, equivalent to a pregnancy rate of 24.9%.

As for endometrial cancer, the National Comprehensive Cancer Network (NCCN) 2012 guidelines for uterine neoplasms affirm that although hysterectomy and appropriate staging is the recommended treatment for endometrial cancer, initial hormonal therapy can be considered for young women with either atypical endometrial hyperplasia or grade I endometrial carcinoma, and who desire fertility preservation [21]. However, their ultimate recurrence rate remains high (around 44%) and the disease progression rate is around 5 to 6% [22]. Moreover, the ACOG 2005 bulletin considers that progestational agents may be a treatment option for selected candidates, but that the disease will likely recur in most patients, which requires the need of continued histologic monitoring, approximately every 3 months [23]. Many questions still remain unanswered, such as ideal progestin regimen, duration of treatment, predictive criteria for responsiveness, and need or timing for completion hysterectomy after childbearing, to cite only few [24]. In a multicenter Korean prospective study of hormonal treatment, cure rate for endometrial carcinoma was 55% and recurrence rate was 57% [25].

Conception is the ultimate goal of fertility-sparing treatment and reproductive outcomes should be one of our main concerns.

The majority of young patients with endometrial carcinomas tend to have coincident polycystic ovary syndrome or ovulatory disorders further hindering their fertility. Contrariwise, in our case, the patient conceived spontaneously. Furthermore, high dose progestin treatment and repeated curettages could also lead to atrophy and uterine synechia [16]. It is worthy to note that endometrectomy was performed in our patient. This did not alter the possibility of conceiving. No synechia were noted on consecutive hysteroscopies. In the literature, only two studies have proposed endometrectomy in the management of endometrial carcinoma [26,27]. This may reduce the bulk of the disease and accelerate the cure. Patients should probably be counseled in order to attempt immediate pregnancy after conservative treatment, and to use assisted reproductive techniques (ART) more readily, especially with the absence of current evidence that ovulation induction agents are associated with a higher risk of recurrence [15]. Most of the case reports and retrospective series report abundant use of ART for achieving pregnancy, mainly because of the short time frame [17].

As for ovarian cancer, no relevant reproductive impairment exists, with data concerning women who attempted conception after FSS reporting a 66 to 100% success rate [1].

It is worthy to note here that our patient received adjuvant chemotherapy along with progestin therapy, in accordance with numerous studies demonstrating the usefulness of adjuvant chemotherapy in increasing the disease-free and overall survival rates in early-stage ovarian cancers [28].

To our knowledge, despite the abundant literature on synchronous ovarian and endometrial tumors, and the numerous reports on conservative treatment conducted for each one of these tumors, this is the first attempted case of fertility sparing surgery for synchronous tumors, and it resulted in a spontaneous pregnancy and normal delivery, which further demonstrates its uniqueness.

## Consent statement

Written informed consent was obtained from the patient for publication of this case report.

## Abbreviations

ACOG: American college of obstetrics and gynecology; ART: Assisted reproductive techniques; EOC: Epithelial ovarian cancer; ESMO: European society for medical oncology; FSS: Fertility-sparing surgery; NCCN: National comprehensive cancer network.

## Competing interests

The authors declare that they have no competing interests.

## Authors’ contributions

DA participated in the care of the patient and wrote the article. RR participated in the care of the patient. JS and NK: participated in the writing of article. GC validated content and form of the article. All authors read and approved the final manuscript.
